# Efficacy of regional arterial embolization before pleuropulmonary resection in 32 patients with tuberculosis-destroyed lung

**DOI:** 10.1186/s12890-018-0722-5

**Published:** 2018-10-01

**Authors:** Gang Chen, Fang-Ming Zhong, Xu-Dong Xu, Guo-Can Yu, Peng-Fei Zhu

**Affiliations:** Department of Thoracic Surgery, Tuberculosis Surgery, Hangzhou Red Cross Hospital, No. 208 Huancheng East Road, Xiacheng District, Hangzhou, 310003 Zhejiang China

**Keywords:** Tuberculous destroyed lung, Pleuropulmonary resection, Regional arterial embolization

## Abstract

**Background:**

Treatment of tuberculous-destroyed lung (TDL) with pleuropulmonary resection is challenging. Pulmonary hemorrhage is a frequent complication of this surgical procedure. Continuous efforts have been made to investigate clinical procedures that may reduce intraoperative bleeding effectively. In this study, we evaluated the feasibility and safety of regional arterial embolization before pleuropulmonary resection in patients with TDL.

**Methods:**

The clinical data of 32 patients with TDL were retrospectively reviewed and analyzed. These patients were admitted to the hospital between July 2009 and November 2016. All of the patients had moderate to massive hemoptysis and received regional arterial embolization in affected areas. Then, these patients underwent pleuropulmonary resection within 1 week to 2 months after embolization.

**Results:**

The results showed that 25 patients (78.1%) had bronchial artery, and all patients had non-bronchial systemic artery found in affected areas. Mild to moderate chest pain was reported in 6 patients, and fever was reported in 2 patients. Intraoperative blood loss during pleuropulmonary resection in patients who had received preoperative regional arterial embolization was 625.6 ± 352.6 ml. Duration of the operation was 120.3 ± 75.2 min. Bronchopleural fistulae and empyema were found in 3 cases (9.4%).

**Conclusion:**

Performance of regional arterial embolization before pleuropulmonary resection offers a safe and feasible option that reduces intraoperative blood loss and shortens operative time in patients with TDL.

## Impact statement

Performance of regional arterial embolization before pleuropulmonary resection appeared to be a safe and feasible option that reduced intraoperative bleeding volume and shortened duration of operation in patients with TDL.

## Background

Tuberculosis (TB) may cause extensive destruction of the lung and compromise lung function. Pathological changes such as extensive fibrosis, bronchial stenosis, and bronchiectasis are commonly observed in the TB-destroyed lung (TDL) [1]. Erosion of a pulmonary artery branch due to TDL can cause bleeding, resulting in gas exchange disorder or sometimes a life-threatening situation [2]. Management options of TDL include the use of anti-TB agents (for active-TB cases), antibiotics (for inactive-TB cases), or surgical intervention. Unfortunately, the prevalence of multidrug resistance (MDR) is high [3]. Bronchial artery embolization (BAE) works in some patients, but a high recurrence rate prevents its widespread application in clinical practice [4]. In the light of this, surgical resection is an option for consideration.

The most common surgical procedure for patients with TDL is pleuropulmonary resection [2, 5–7]. TDL affected areas are usually extensive with severe adhesions. In addition, they are accompanied with recurrent infections, abundant collateral circulation, and hemorrhage. Complications are likely to occur during the procedure of separation in conventional pleuropulmonary resection of TDL. Homeostasis is difficult to maintain during the operation [8]. To minimize the complication rate, reducing intraoperative blood loss is an important factor to consider. Recently, our hospital has adopted a procedure of regional arterial embolization in combination with pleuropulmonary resection, in order to reduce intraoperative and postoperative bleeding. Thirty-two TDL patients with massive hemoptysis received regional arterial embolization before pleuropulmonary resection between July 2009 and November 2015. We retrospectively analyzed these cases and evaluated the efficacy of regional arterial embolization in combination with pleuropulmonary resection. The results may provide insight into the development of new treatment options for patients with TDL.

## Methods

### Baseline information

A total of 32 patients with TDL were included in this study. The disease duration ranged from 30 to 240 months, with a mean of 56.7 months. All patients received a standard or other non-standard preoperative anti-tuberculosis therapy. Demographics (gender and age), disease duration, location of the destroyed lung and indications are shown in Table 1. Tuberculosis was diagnosed by pathological or etiological examination. The diagnostic criteria included: 1) positive acid-fast bacilli in respiratory specimens (e.g. sputum, bronchoscopy lavage fluid, surgically resected lung tissue) by smear or culture; 2) PCR-positive for *Mycobacterium tuberculosis* RNA in respiratory specimens; 3) caseating granuloma found in the histopathological examination of surgically resected lung tissue. The ethics review board of Hangzhou Red Cross Hospital approved this study. Surgeons, who had legal eligibility for interventional treatment and extensive experience in tuberculosis thoracic surgery, conducted all interventional procedures and operations.

**Table 1:** Demographics, indications and locations of destroyed lung in patients

Clinical features	Results
Demographics	
Gender	
Male (n)	21(65.6%)
Female (n)	11(34.4%)
Age (years)	
Range	25–69
Mean ± SD	37.8 ± 11.2
Duration of disease (month)	
Mean ± SD	56.7 ± 26.5
Locations of destroyed lung (n)	
Unilateral lung	20(62.5%)
Single upper lobe	9(28.1%)
Middle or lower lobe	3(9.4%)
Indications (n)	
Hemoptysis	32(100%)
Continuously positive smear	9(28.1%)
Pulmonary aspergillosis	13(40.6%)

### Criteria for diagnosis and inclusion

We defined patients with TDL as those presenting with a destroyed lung with a history of tuberculosis (diagnosed by physician with proof of positive smears/cultures). Computed tomography (CT) scan was performed to examine the lesions of parenchymal destruction. All patients included in this study fulfilled the eligibility criteria. The inclusion criteria were as follows: (a) TDL affected areas in at least one lung lobe; (b) history of moderate to massive hemoptysis (amount of hemoptysis was greater than 100 mL within 24 h); (c) repeated episodes of cough or hemoptysis after conventional treatment (e.g. first-line/second-line anti-tuberculosis drugs, intravenous and oral hemostatic drugs, vascular embolization). Exclusion criteria were as follows: (a) incomplete clinical information; (b) history of thoracic surgery; (c) active tuberculosis without standardized anti-tuberculosis medical treatment; (d) uncontrolled concomitant diseases such as heart failure, asthma, idiopathic pulmonary fibrosis, and malignant tumor.

### Preoperative preparation

All patients received CT scan, bronchoscopy, routine blood test, heart and lung function tests, sputum TB smear/culture, and drug sensitivity test before the surgery.

Preoperative fiberoptic bronchoscopy was used to confirm absence of active endobronchial tuberculosis in all cases. Patients received standard anti-tuberculosis treatment for at least 6 months. Thereafter, patients who still had acid-fast bacilli-positive sputum smear were given second-line anti-tuberculosis treatment (sodium aminosalicylate, amikacin, ethionamide and levofloxacin) for at least 3 months. Those with signs of pulmonary aspergillosis in radiological images received preoperative itraconazole or voriconazole for at least 1 month.

Arterial supply of lesions was preliminarily identified by multislice spiral CT (MSCT). Then, embolization of regional systemic arteries was performed at the interventional center. Finally, pleuropulmonary resection was performed in the operation room within 1 week to 2 months.

All patients were followed up for 6 to 48 months, and reports of complications were collected during outpatient visits or subsequent readmission.

### Regional arterial embolization

Arterial supply to the affected areas was examined by MSCT. A transfemoral artery puncture was next made. Cordis 4F/5F C2 or VER135 angiographic catheter, Merit Meastro 28MC24130SN microcatheter were used in the surgery. Digital subtraction images were taken after injection of iodinated contrast agent. Particle embolization was performed as distal as possible in order to avoid embolization of adjacent spinal arteries. A mixture of 500–700 m Particle Embolic Agent (PVA) or gelatin sponge particles and the diluted contrast agent were used as the embolic material for peripheral arteries. Short acting embolic agents such as gelatin sponge were used in the surgical areas, and permanent embolic agents such as PVA were used in areas out of the planned surgical site. Gelatin sponge was used as an embolic material for main vessels. Blood supply to the main bronchus area was preserved. Lastly, the catheters were removed and the puncture sites were pressurized for hemostasis. The duration of embolization ranged from 30 min to 120 min, with a mean of 45 min. If postoperative complications occurred, treatment was given immediately to relieve the symptoms.

### Pleuropulmonary resection

Patients were placed in the contralateral decubitus position and underwent general anesthesia. Incisions of 5–10 cm were made at the 4th or 5th intercostal space between the mid-axillary lines, and separations were performed. If possible, the pulmonary hilum was treated with priority, as it may reduce the amount of bleeding during the operation. When it is difficult to separate the pulmonary vessels, a direct incision of the pericardial sac may be made. The bronchial stump was sutured with an automatic suturing device,then with a 3–0 free suture. Argon knife treatment was usedfor the bleeding wound. After a complete hemostasis was reached, thoracic cavity was repeatedly flushed with warm saline. Thoracic cavity was closedwith standard procedure.

### Statistical analysis

Data were shown as mean ± standard deviation (SD). All data were analyzed by SPSS 23.0 statistical software packages.

## Results

The results of interventional angiography showed that 25 cases (78.1%) had bronchial artery, with pathological changes, in the affected areas. The non-bronchial systemic artery (NBSA) was noted in all patients: 26 cases (81.3%) with posterior intercostal arteries, 5 cases (15.6%) with internal thoracic arteries, 11 cases (34.4%) with external thoracic arteries, 9 cases (28.1%) with subclavian arteries, and 8 cases (25.0%) with inferior phrenic arteries.

Of the 32 patients, 20 patients received pleuropneumonectomy, 12 patients received pleurolobectomy (upper lobe resection in 7 cases, lower lobe resection in 3 cases, upper lobe resection and segmentectomy in 2 cases). After preoperative regional arterial embolization, the estimated intraoperative blood loss was 625.6 ± 352.6 mL, and the operative time was 120.3 ± 75.2 min. Six patients received 3–5 h of postoperative mechanical ventilation in the ICU.

All the regional arterial embolization and pleuropulmonary resections were successfully completed. All cases were diagnosed with pulmonary tuberculosis. No death was reported.

Complications of the interventional therapy were reported in 8 cases. These included 6 cases of mild to moderate thoracodynia, which were relieved after treatment with non-steroidal analgesics and 2 cases of fever, which were relieved after treatment with antibiotics and antipyretic. Renal failure, paralysis and other complications were not observed.

Postoperative complications were found in 6 cases. These included 3 cases (9.37%) of persistent pulmonary air leak, of which 2 cases fully recovered by continuous suction drainage, and 1 case was treated with open thoracic drainage after 6 months (the patient underwent continuous closed chest drainage 6 months after surgery, and pneumothorax was still observed). Postoperative empyema occurred in 3 cases, immediately after surgery in 2 cases, and 1 month postoperatively in 1 case. All of them were cured by continuous thoracic drainage. Safety follow-up was performed for all the 32 patients (range, 6 to 48 months; median, 30 months). Among these patients, five were MDR or mono-resistant, in which 1 was H resistant, 2 were HR resistant, 1 was HRS resistant, and 1 was HRES resistant (H: isoniazid; R: rifampicin; E: ethambutol; S: streptomycin). Chest roentgenogram and sputum bacteriological investigations were conducted in the follow-up visits. The sputum negative conversion rate and the clinical cure rate were 100%.

## Discussion

Tuberculosis can cause extensive destruction of the lung. Expansion ofblood vessels may occur after repeated infections, and pulmonary hemorrhage is a frequent complication of conventional pleuropulmonary resection of TDL. Duan et al. suggested that intraoperative blood loss over 1000 mL and operative time more than 4 h are two important risk factors of postoperative complications [8]. Bai et al. reported a group of 172 patients of TDL, who received surgical treatment, and the operative blood loss reached 1240.0 ± 1122.5 mL [9], suggesting that the intraoperative blood loss was generally large in TDL patients. Therefore, the control of blood loss is important for the success of pleuropulmonary resection of TDL.

In the present study, we found that mediastinal pleural adhesion was relatively loose even though the pleural cavity had dense adhesions. Therefore, we mostly isolated mediastinal adhesions with priority to facilitate exposure and separation of pulmonary vessels. The probability of upper lobe damage was high, which occurred in 29 cases (90.6%) in our study. Adhesion at the upper portion of the pleural cavity was difficult to separate, because of the combined thoracic constriction deformity. In addition, the abnormal blood vessels at the pleural adhesions were very dense; therefore, wound bleeding occurred frequently when the pleura was separated. In our operations, the 4th intercostal approach was taken if the major lesion was in the upper lobe. Vats were used to enlarge the field of vision if necessary. Pale ischemic changes were found in many wounds during the operations. After pre-embolization of regional blood vessels, wound bleeding was significantly reduced during the separation of pleural adhesions. Overall, the arterial embolization procedure appeared to reduce the risk of bleeding, and the duration of operation was shortened in subsequent separation and resection of the lesion.

**Fig. 1:**
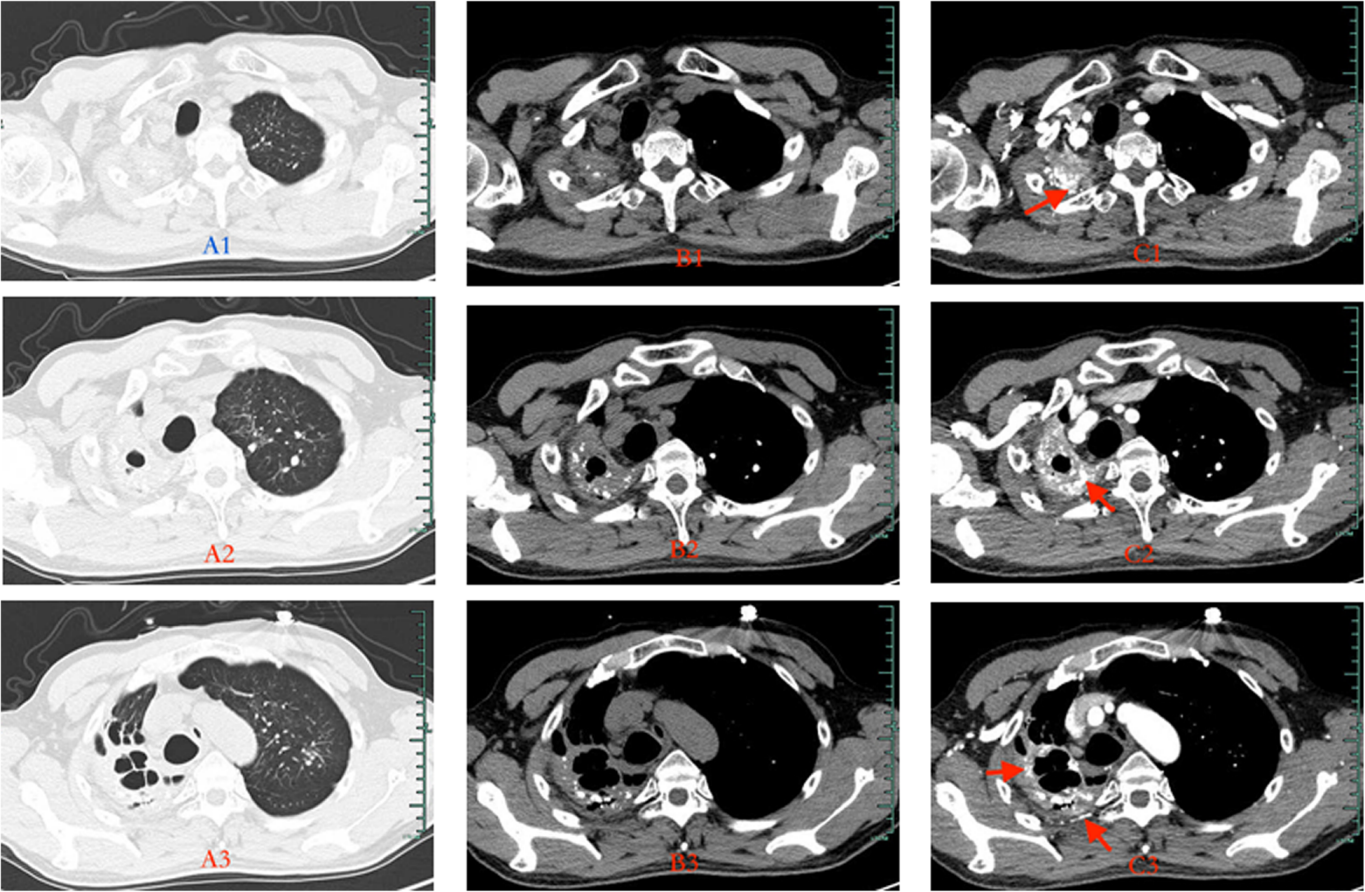
MSCT images taken before regional arterial embolization (male, 58 years old, repeated episodes of hemoptysis for more than 2 years; after two intervention treatments, hemoptysis was still observed, the largest amount of hemoptysis was 500 mL): vascular enhanced CT showed an increased supply of blood at the pleura

NBSA may be more likely to occur in patients with hemoptysis caused by tuberculosis. Jiang et al. reported that hemoptysis in more than half of different cases was related to NBSA [10]. Adhesions formed among the lung, intercostal, subclavian, and the parietal pleura. Repeated infections lead to regional vasodilatation, and collateral circulation becomes more abundant (Fig. 1). In our study, all patients were complicated with NBSA blood supply; 26 cases (81.3%) had posterior intercostal arteries, 5 cases (15.6%) had internal thoracic arteries, 11 cases (34.4%) had external thoracic arteries, 9 cases (28.1%) had subclavian arteries, and 8 cases (25.0%) had inferior phrenic arteries. These types of artery supply caused operative challenges, including increased intraoperative bleeding and longer duration of operation.

**Fig. 2:**
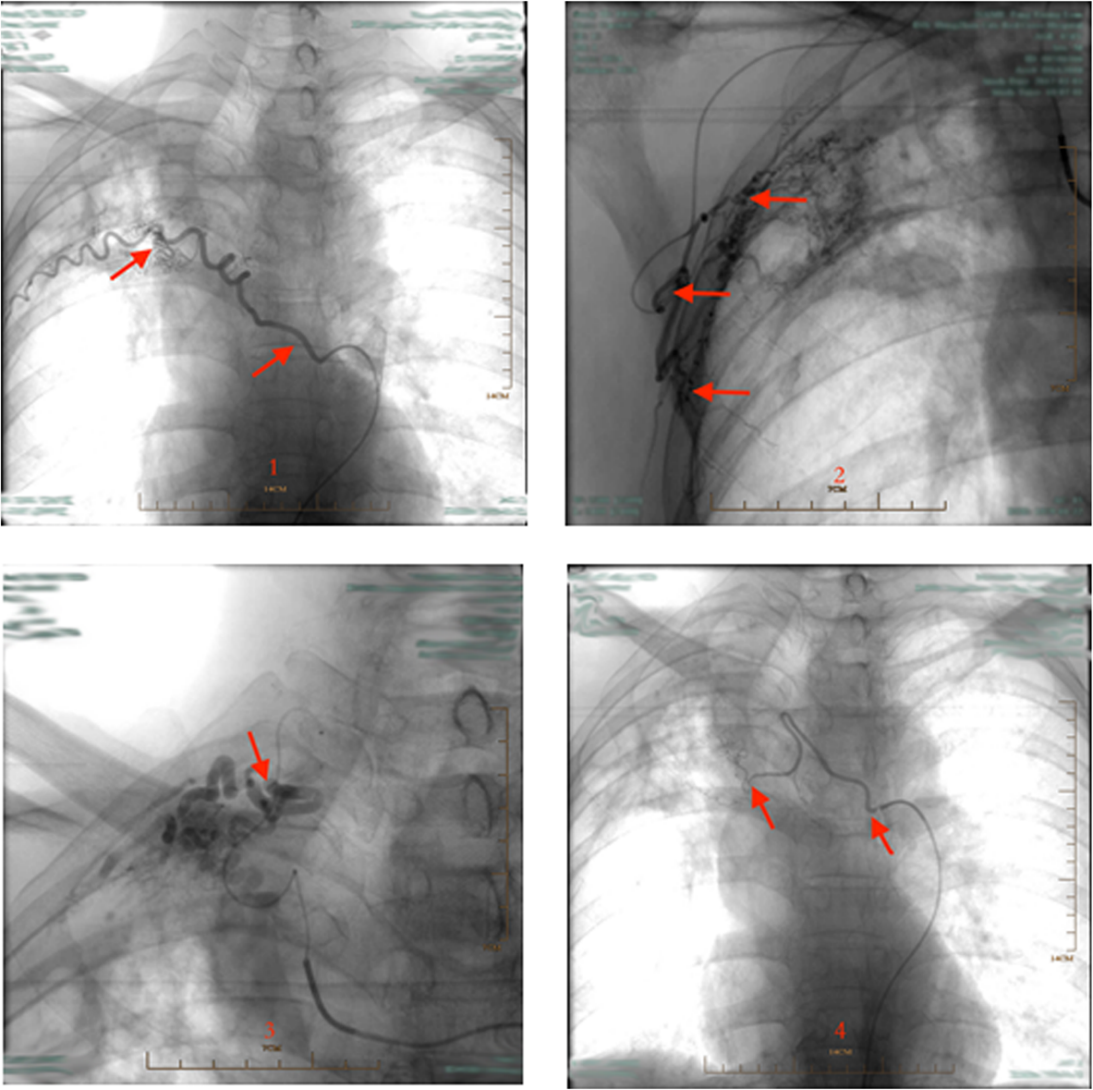
Parts of pathologic arteries in the lesion of TDLs, which were found in the course of interventional therapy (the same case as Fig. 1). 1: pathological posterior intercostal artery; 2: pathological external thoracic artery; 3: pathological subclavian artery; 4: pathological bronchial artery

**Fig. 3:**
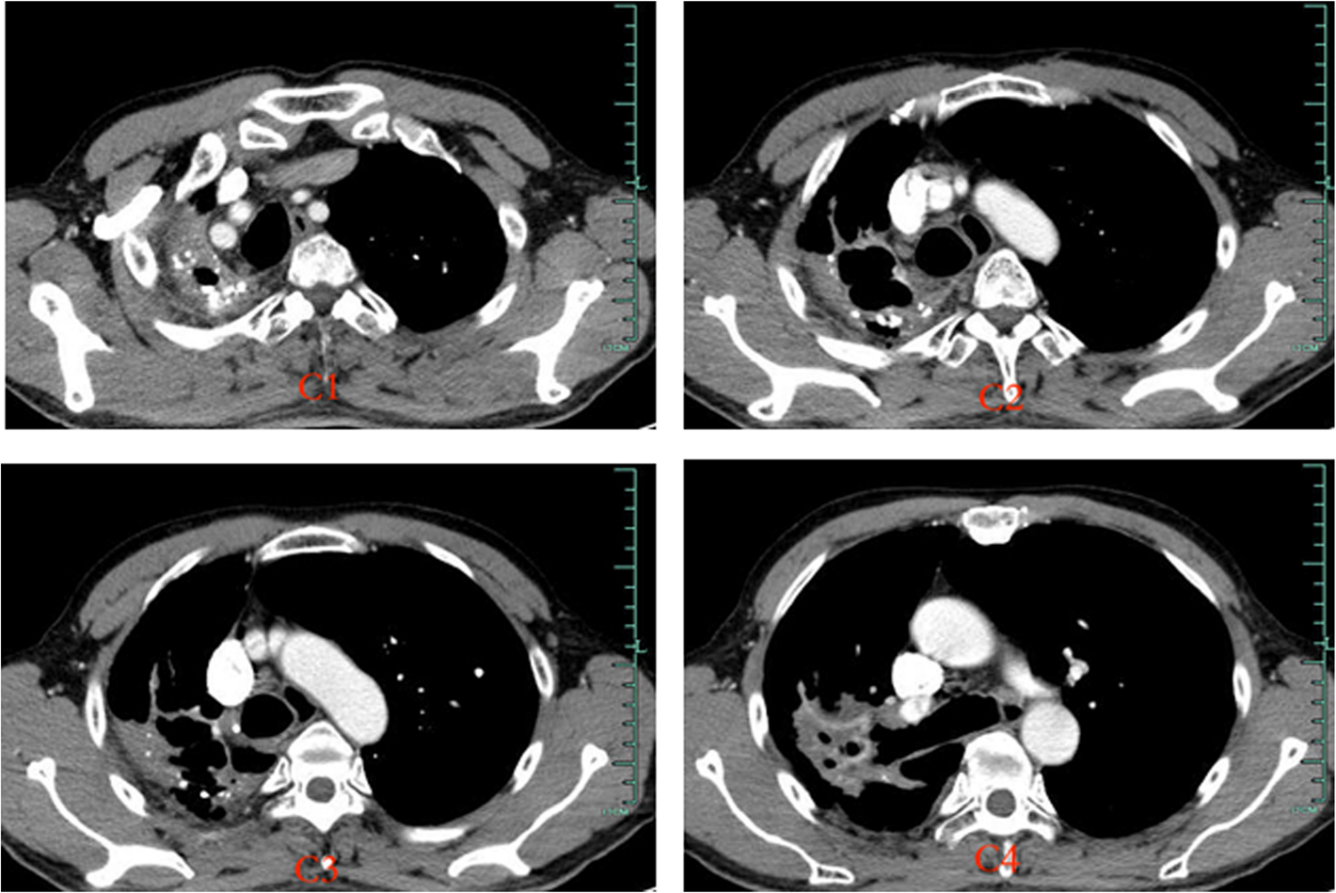
CT images taken 1 month after regional arterial embolization. (The same case as in Fig. 1):vascular enhanced CT showed a marked reduction in blood supply at the pleura, compared with the time before embolization

CT angiography (CTA) is an effective method for accurate evaluation of blood vessel responsible for hemoptysis. It has a high sensitivity for diagnosis of pathological artery [11]. Dual arterial CTA of the bronchial artery and non-bronchial artery can provide better preoperative guidance for percutaneous catheter embolization [12]. In particular, it helps to avoid missing the observation of arteries such as the subclavian artery and the internal thoracic artery. In our department, dual arterial CTA of the BA and the NBSA were arranged to fully assess the distribution of the system arteries in the affected areas. Therefore, the target blood vessel was found and embolized by injecting embolic agents under DSA guidance (Fig. 2). After vascular embolization, abnormal blood vessels at the pleural adhesions were markedly reduced (Fig. 3). Pre-embolization appeared to provide good surgical conditions for subsequent pleuropulmonary resection. It is important to emphasize that the effect of preoperative arterial embolization in the surgical area is temporary. Short acting embolic agents such as gelatin sponge were used, and the vessels in the main bronchus area were preserved. As a result, the blood supply to the bronchial stump was not affected, and the risk of postoperative pleural fistula was minimized. Lastly, vascular embolization should not be too thorough; operation that may damage ectopic vessels should be avoided. Complications such as spinal cord injury and transient dysphagia were not observed in our study.

## Conclusions

The current study has its limitation, for example the sample size was small, and prospective studies are needed to confirm the findings. In addition, patients with TDL without massive hemoptysis were not included because of treatment indications or ethical reasons. In summary, the findings of the current study suggested that regional arterial embolization before pleuropulmonary resection for TDL may reduce the risk of pleuropulmonary resection, shorten the operation time, and reduce postoperative complications. Further study is warranted to investigate if this treatment can be widely adopted in clinical settings for patients with TDL.
